# Maize Resistance to Stem Borers Can Be Modulated by Systemic Maize Responses to Long-Term Stem Tunneling

**DOI:** 10.3389/fpls.2020.627468

**Published:** 2021-03-11

**Authors:** Víctor Manuel Rodríguez, Pablo Velasco, Ana Cao, Rogelio Santiago, Rosa Ana Malvar, Ana Butrón

**Affiliations:** ^1^Misión Biológica de Galicia (CSIC), El Palacio-Salcedo, Pontevedra, Spain; ^2^Departamento de Biología Vegetal y Ciencias del Suelo, Facultad de Biología, Universidad de Vigo, As Lagoas Marcosende, Agrobiología Ambiental, Calidad de Suelos y Plantas (UVIGO), Unidad Asociada a la MBG (CSIC), Vigo, Spain

**Keywords:** induced resistance, antibiosis, metabolomic response, *Sesamia nonagrioides*, stem corn borer

## Abstract

Limited attention has been paid to maize (*Zea mays* L.) resistance induced by corn borer damage, although evidence shows that induced defenses have lower resource allocation costs than constitutive defenses. Maize responses to short- and long-term feeding by the Mediterranean corn borer (MCB, *Sesamia nionagrioides*) have been previously studied, but the suggested differences between responses could be due to experimental differences. Therefore, in the current study, a direct comparison between short- and long-term responses has been made. The objectives were (i) to determine changes in the level of antibiosis of the stems induced by feeding of *S. nonagrioides* larvae for 2days (short-term feeding) and 9days (long-term feeding), (ii) to characterize the metabolome of the stems’ short- and long-term responses to borer feeding, and (iii) to look for metabolic pathways that could modulate plant resistance to MCB. Defenses were progressively induced in the resistant inbred, and constitutive defenses were broken down in the susceptible inbred. Results suggest that the different resistance levels of the two inbreds to stem tunneling by MCB could depend on their ability to establish a systemic response. Based on these results, a high throughput look for specific metabolites implicated in systemic induced resistance to maize stem borers is recommended; the current focus on constitutive defense metabolites has not been successful in finding molecules that would be valuable tools for pest control.

## Introduction

Stem tunneling by maize stem borers is an important constraint to achieve the potential yield of maize varieties across the world (Malvar et al., 2008). The maize plants protect themselves from borer feeding using constitutive and induced defenses. Much attention has focused on studying the former, although evidence indicates that induced defenses have lower resource allocation costs than constitutive defenses (Klenke et al., 1986; Beeghly et al., 1997; Karban et al., 1997; Cardinal and Lee, 2005; Santiago et al., 2006a,b, 2011; Howe and Jander, 2008). Indeed, maize stem feeding by borers significantly modifies antibiosis against stem borer larvae. However, these changes depend on the genotype and the duration of feeding. We consider plant responses to 1 or 2days of feeding by herbivores as short-term responses; meanwhile, long-term responses will be generated after more than 1week of continuous feeding. Dafoe et al. (2013) reported that the growth of stem borer larvae was significantly higher when fed with stem tissues preconditioned by 48h of larval tunneling compared to untreated stem tissues, while Cao et al. (2019) stated that the effect of long-term feeding by borers on the antibiotic properties of corn stems is genotype-dependent.

Previous studies have already shown that feeding-induced changes in plant metabolites influence the behavior and performance of conspecific herbivores, and that influence depends on the time lag of induction (Poelman et al., 2008; Wang et al., 2015; Santiago et al., 2017; Su et al., 2018). In that regard, several authors have pointed out specific defense mechanisms involved in the response to long-term feeding by insects (Gutsche et al., 2009; Uawisetwathana et al., 2015; Donze-Reiner et al., 2017). In the particular case of maize, important differences between maize stem responses to short- and long-term feeding by stem borers have been reported (Dafoe et al., 2011; Rodriguez et al., 2012, 2018). The early stem response to feeding by corn borers was characterized by the activation of signaling mechanisms mediated by phytohormones, whereas these molecules were only marginally involved in the long-term response (Dafoe et al., 2011; Rodriguez et al., 2012). The stem’s long-term response was characterized by reorganization of the primary metabolism and a strong redox response, mainly mediated by germin-like proteins to produce anti-nutritive and toxic compounds that reduced insect viability (Rodriguez et al., 2018). However, no studies have simultaneously characterized short- and long-term responses to stem feeding by stem borers, and the current work would be the first attempt to do so. A direct comparison of both responses using several genotypes under the same experimental conditions will shed light on real differences, which cannot be disentangled from experimental differences when results from different experiments are compared. The stem borer species selected to perform this direct comparison was *Sesamia nonagrioides* Lef. [the Mediterranean stem borer that is often called Mediterranean corn borer (MCB)]. The MCB shows two to four generations per year; larvae of the first generation being scarce, feeding on the whorl of juvenile plants, and causing plant death or delayed development; meanwhile, subsequent generation larvae are more numerous and feed preferentially on the stem pith of adult plants (Cordero et al., 1998).

Since metabolome can be viewed as the end product of gene expression, un-targeted metabolomics would be a valuable tool to monitor the biological processes operating in the plant response to herbivory (Sumner et al., 2003; Schauer and Fernie, 2006; Jansen et al., 2009). Aside from a direct plant defense, herbivory could induce extensive metabolic changes to prevent the allocation of energy and nutrients to herbivore fitness. Plant metabolites involved in defense are not just toxic, repellent, and/or anti-nutritive molecules but compounds that could attract enemies of herbivores that could participate in nutrient transport and storage to make nutrients less accessible to the insect or that are involved in phenology shifts that grant herbivore avoidance (Schuman and Baldwin, 2016).

The objectives of this work were (i) to determine changes in the level of antibiosis of the stems induced by feeding of MCB larvae for 2days (short-term feeding) and 9days (long-term feeding), (ii) to characterize the metabolome of the stems’ short- and long-term responses to borer feeding, and (iii) to look for metabolic pathways that could modulate plant resistance to MCB.

## Materials and Methods

### Plant Materials and Treatment Applications

**Figure 1:**
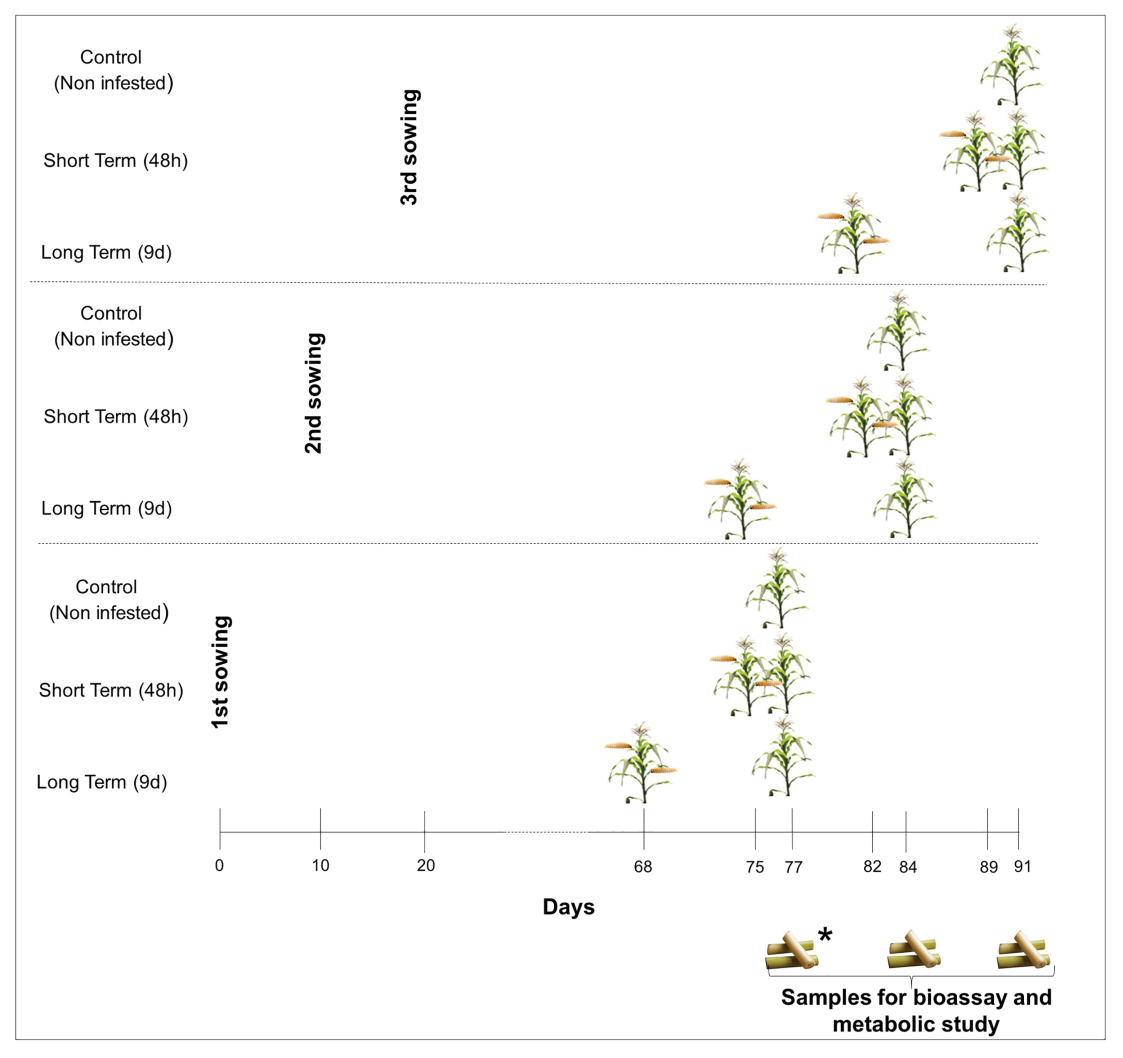
Greenhouse experiment design. ^*^Maize samples for establishment of the bioassay and metabolite characterization were taken 77days after planting in the earliest planting. Maize tissues from the second and third plantings were taken at 84 and 91days, respectively, to renew feeds for Mediterranean corn borer (MCB) larvae.

Two different maize inbred lines (PB130 and EP42) were used as plant material. In previous works, we classified these inbred lines as resistant (PB130) and susceptible (EP42) to MCB feeding at maturity stage (R6) and significantly different in their early response to stem tunneling by MCB larvae (Butron et al., 1998, 1999; Rodriguez et al., 2012). Maize plants were individually grown, under greenhouse conditions, in 10-L pots filled with peat and fertilized according to maize needs. We performed three consecutive plantings separated by 10days to guarantee that, during the bioassay, MCB larvae were always fed with stems from plants around tasseling stage. In the earliest planting and within each genotype, 10 plants were infested 9days before the establishment of the bioassay by placing 2second-instar larvae between the stalk and the sheath of basal leaves, 10 plants were infested 48h before, and 10 plants were left untreated (control). Infested plants were protected with nets to avoid larval dispersion to non-infested pots. Since stems to feed the larvae were renewed during the bioassay, treatments were also done as described above in the second and third planting dates to guarantee that stem portions given to the larvae came from plants around the tasseling stage (Figure 1). Plant tissue at tasseling was chosen because second and subsequent generation larvae can attack maize from tasseling to harvest time but plants at earlier stages of development are more susceptible to damage by *S. nonagrioides* (Ordas et al., 2013).

### Non-choice Feeding Bioassays

To study the effect of short- and long-term feeding, MCB larvae were fed with stems of plants infested for 48h (short-term feeding), plants infested for 9days (long-term feeding), or non-infested plants before bioassay establishment. Based on the biology and voraciousness of this species, feeding periods for studying short- and long-term responses to stem feeding were established as 2- and 9-day feeding times, respectively, because we wanted to guarantee that the pith is damaged but at levels that do not compromise the availability of undamaged stem tissue for analysis. Therefore, 2-day feeding was used for studying the short-term response because larvae would need 48h to penetrate the stem and start to feed on the pith; meanwhile, as this species has great voracity, a 9-day feeding period would render large damage to stems but without compromising the disposal of undamaged tissue.

First-instar MCB larvae were initially weighed and individually placed in plastic tubes on 2-cm sections of the stem portion that goes from the ground to main ear insertion node. Larvae were previously fed on a maize-based artificial diet for 48h and maintained at starvation for 24h, presenting weights of 1–3mg at the beginning of the bioassay. Sixty larvae per treatment and genotype were set and maintained in a growth chamber under controlled conditions of temperature and humidity [22°C, 80% relative humidity (RH)] and a photoperiod of 16L:8D. When necessary, new fresh stem portions of plants (control or preconditioned by stem feeding during 2 or 9days) were provided to the bioassay larvae. Renewal of stem cuts was done weekly at the beginning of the bioassay and every 3–4days at the end of the bioassay to prevent undesirable effects of tissue deterioration on larval development and to avoid larval starvation when larvae began to increase tissue consumption. Larval weights and data related to dead larvae were recorded at 7, 11, 14, 18, 22, and 26days after bioassay establishment.

A repeated-measures analysis was performed to test differences for larval weights using the PROC GLIMMIX procedure of SAS software (SAS, 2008; Stroup, 2013). Initial larval weight was included as a covariate, genotype and treatment were set as fixed factors and a first-order autoregressive covariance structure (AR-1) was chosen in the within-subject correlation. Within each genotype, differences for larval weight of treatments were tested at each time using least square (LS) means, adjusted by the initial larval weight. Additionally, linear and quadratic coefficients of regression of larval weight on time were obtained for each treatment-genotype combination. Within each genotype, the comparison of the larval growth curves of treatments was performed by making orthogonal contrasts among treatment regression parameters (intercept, linear and quadratic components, respectively; *p* ≤ 0.05; Littell et al., 2006). The PROC LIFETEST procedure of SAS software was used to test differences of larval survival among treatments applied to the same genotype using the Kaplan-Meier method (SAS, 2008). The death of larvae was the event of interest, and the missing and live larvae at the end of the bioassay were treated as censored data. The homogeneity of the survival distributions was tested using the Šidák multiple-comparison adjustment for log-rank test (*p* ≤ 0.05).

### Metabolomics Characterization of Maize Responses to Mediterranean Corn Borer Feeding

Metabolomic profiles were obtained in five biological replicates per genotype-treatment combination. A 3-cm portion at the bottom of the stem internode below the ear was taken from each plant. Frozen samples were lyophilized and ground to a fine powder using an electric mill. Metabolites were extracted using 50mg of this powder through 500μl of 80% aqueous methanol by sonication for 15min. Samples were centrifuged for 10min in order to remove plant debris (16,000 × *g*, at room temperature). Supernatant was filtered through a 0.20-μm polytetrafluoroethylene (PTFE) micropore membrane and placed in vials for further analysis. Five microliters of each sample were injected into an ultra-high-performance liquid chromatography (UHPLC) system (Thermo Dionex Ultimate 3000 LC) connected to a QTOF detector (Bruker Compact™) with a heated electrospray ionization (ESI) source. Chromatographic separation was performed in an Intensity Solo 2 C18 column (2.1mm × 100mm, 1.7μm pore size; Bruker Daltonics, Germany) using a binary gradient solvent mode consisting of 0.1% formic acid in water (solvent A) and acetonitrile (solvent B). The following gradient was used: 3% B (0–3min), from 3 to 25% B (3–10min), from 25 to 80% B (10–18min), from 80 to 100% B (18–22min), then held at 100% B until 24min. The flow rate was established at 0.4mlmin^−1^, and column temperature was controlled at 35°C.

Mass spectrometry (MS) data were acquired using an acquisition rate of 2Hz over the mass range of 50–1,200 m/z. Both polarities (±) of ESI mode were used under the following specific conditions: gas flow 9Lmin^−1^; nebulizer pressure 2.6bar; dry gas 9Lmin^−1^; dry temperature 220°C. Capillary and endplate offsets were set to 4,500 and 500V, respectively. To monitor the performance of data acquisition, the run sequence was started with three blanks (methanol, the solvent used in sample extraction) and a standard compound (triphenyl phosphate in positive ionization mode and chloramphenicol in negative ionization mode). Auto MS/MS fragmentation in pooled samples was performed in order to facilitate compound identification. For MS/MS analysis, data were acquired using an acquisition rate of 8Hz and precursor ions collected using an absolute threshold of 1,500 counts and a cycle time of 1.0s.

The algorithm T-Rex 3D from the MetaboScape 4.0 software (Bruker Daltoniks, Germany) was used for peak alignment and detection. The generated dataset was imported into MetaboAnalyst 4.0 (Chong et al., 2019) to perform statistical analyses. In order to remove non-informative variables, data were filtered using the interquartile range (IQR) filter. Moreover, Pareto variance scaling was used to remove the offsets and adjust the importance of high- and low-abundance ions to an equal level. The resulting three-dimensional matrix (peak indices, samples, and variables) was further subjected to multivariate data analysis. Within each inbred, partial least squares discriminant analysis (PLS-DA) was carried out to investigate and visualize the pattern of metabolite changes between the control and each infestation treatment. This analysis was applied to obtain an overview of the complete dataset and discriminate those variables that are responsible for variations between groups (control vs. 48h feeding to characterize the short-term response to MCB feeding and control vs. 9days feeding to characterize the long-term response). The PLS-DA model was evaluated through a cross-validation (R2 and Q2 parameters). The quality assessment (Q2) and R2 statistics provide a qualitative measure of consistency between the predicted and original data or, in other words, estimates the predictive ability of the model.

For each inbred and infestation treatment, features with a variable importance in projection (VIP) score >2 in the PLS-DA model (control vs. infestation treatment) were selected and considered the most influential features in that inbred response to MCB feeding. Features with a VIP score >2 were retained. For tentative identification, a consensus molecular formula was assigned to each molecular feature based on exact mass data and isotopic pattern distributions for the precursor using MetaboScape 4.0 and Sirius v4 (Dührkop et al., 2019) software. Molecular formula was used to perform identification analysis on publicly available databases: PubChem (Kim et al., 2019), MassBank (Horai et al., 2010), Kyoto Encyclopedia of Genes and Genomes (KEGG) (Kanehisa et al., 2016), KNApSAcK (Afendi et al., 2012), Metlin (Guijas et al., 2018), and Chemspider (Ayers, 2012). When available, the ms/ms fragmentation spectrum of reference compounds identified on databases was compared to that obtained experimentally.

## Results

**Figure 2:**
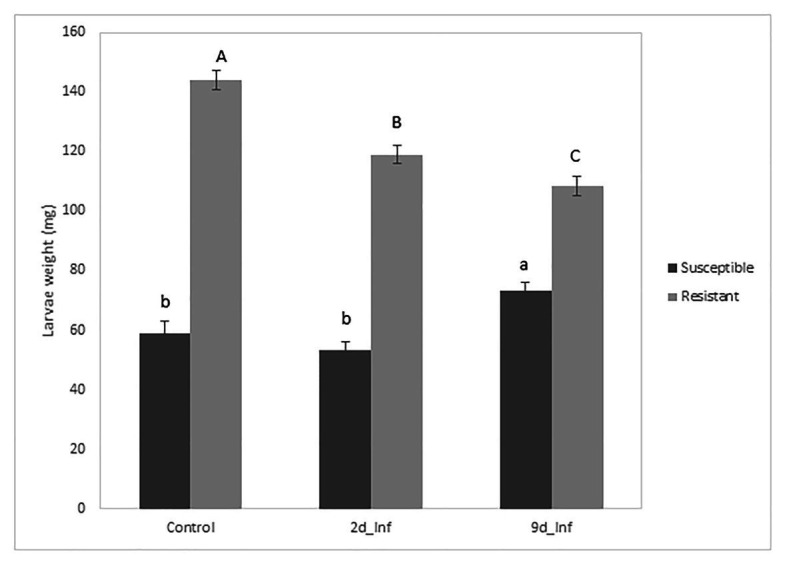
Means of treatments applied to two inbreds for MCB larval weight at the end of the bioassay. Mean comparison among treatments was separately made for each inbred (uppercase letters for the resistant inbred and lowercase letters for the susceptible inbred).

**Figure 3:**
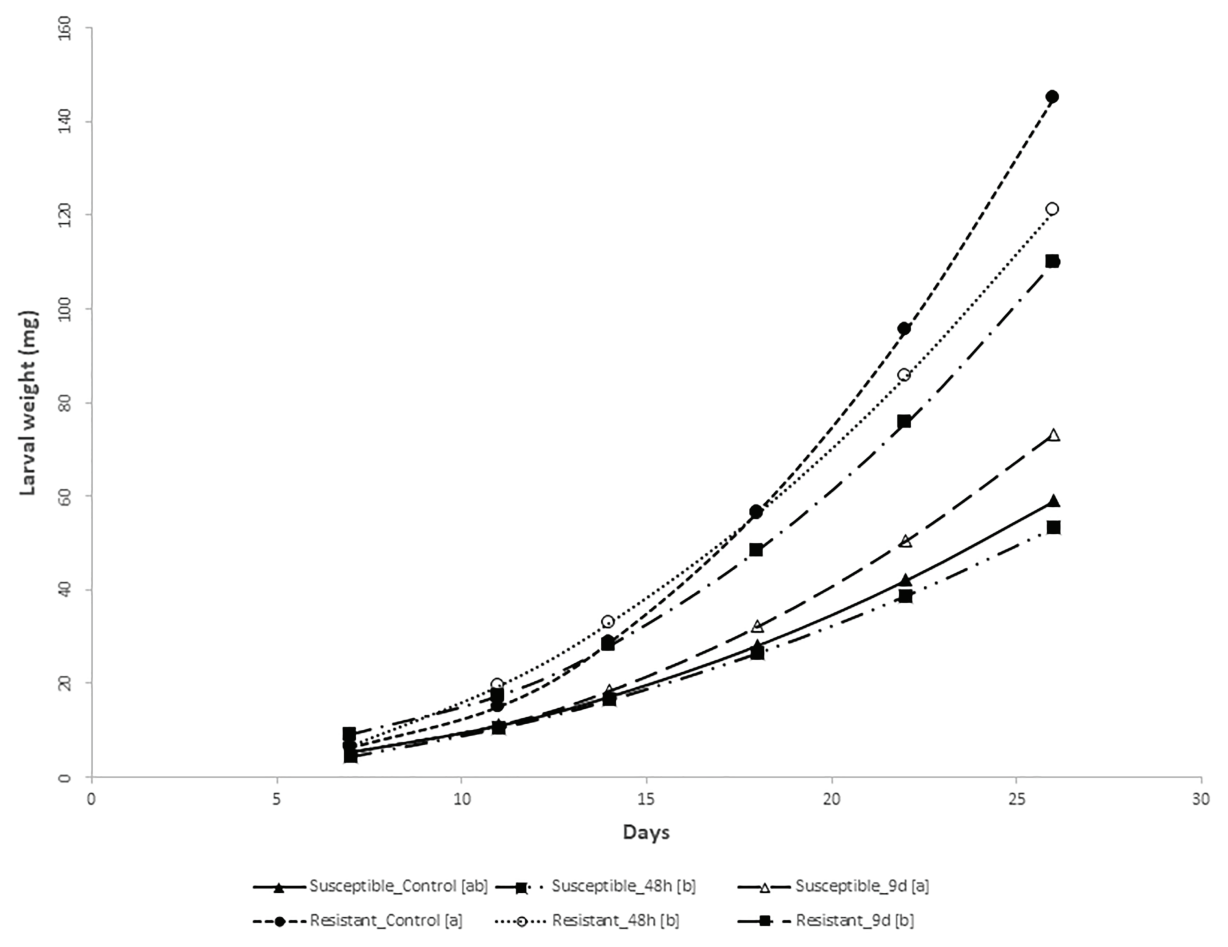
MCB larval growth on non-choice feeding bioassay. Regression curves (including lineal and/or quadratic coefficients) of the weight (mg) of the MCB larvae fed on stems from the resistant and susceptible inbred plants pre-infested with MCB larvae for 48h or 9days (9d) before or untreated (control) on time. Within each inbred, treatments followed by different letters within the brackets [] showed regression curves significantly different based on differences between linear or/and quadratic coefficients.

The analysis of variance for larval weight evolution over 26days showed that all sources of variation (fixed effects and random effects, i.e., first-order autoregressive covariance structure of within-subject correlation among weights recorded on the same larva where *σ*^2^ = 0.7539 ± 0.0163) were significant (*p* < 0.05) except treatments. However, the interaction treatment × genotype was significant, suggesting that maize genotypes differentially respond to feeding treatments. At the end of the bioassay, larvae reared on stems from control susceptible inbred plants weighed less than larvae fed on the resistant ones (Figure 2). Resistant plants preconditioned by 48h of feeding by MCB larvae decreased the larval weight significantly compared to its control, while the average weight of larvae fed with susceptible plants preconditioned by 48h feeding did not differ from the corresponding control larval weight (Figures 2, 3). On the other hand, larvae fed on susceptible plants preconditioned for a longer period (9days) show increased weight compared to the control, whereas the opposite effect was observed on larvae fed on resistant plants. In addition, the mortality of larvae fed on the resistant plants was significantly higher than that of larvae fed on the susceptible ones. No differences in mortality between both inbreds were detected at control conditions (Table 1).

**Table 1:** MCB larval survival on non-choice feeding bioassay.

Log-rank for treatments^a^			Log-rank for inbreds^b^			
Treatment	Resistant^a^	Susceptible	Inbred	Control	48h	9d
Control	0.14 (27%)a	1.92 (23%)a	Resistant	0.95a	2.06a	6.40a
48h	−2.28 (27%)a	1.05 (22%)a	Susceptible	−0.95a	−2.06a	−6.40b
9d	2.01 (33%)a	−2.97 (17%)a				

Values of the log-rank statistic for testing homogeneity of survival distribution of Mediterranean corn borer (MCB) when larvae were reared on stems of plants infested with MCB larvae for 9days (9d) or 48h before and on untreated (control) plants. Positive values of the log-rank statistic indicate that the number of dead larvae is over the number expected under the null hypotheses of equivalent survival distributions.

a Log-rank statistics followed by the same letter in the same column mean that survival curves of the different treatments within the same genotype were homogeneous (*p* < 0.05). The percentage shown between brackets is the mortality percentage.

b Log-rank statistics followed by the same letter in the same column mean that survival curves were homogeneous between genotypes under the same treatment (*p* < 0.05).

**Figure 4:**
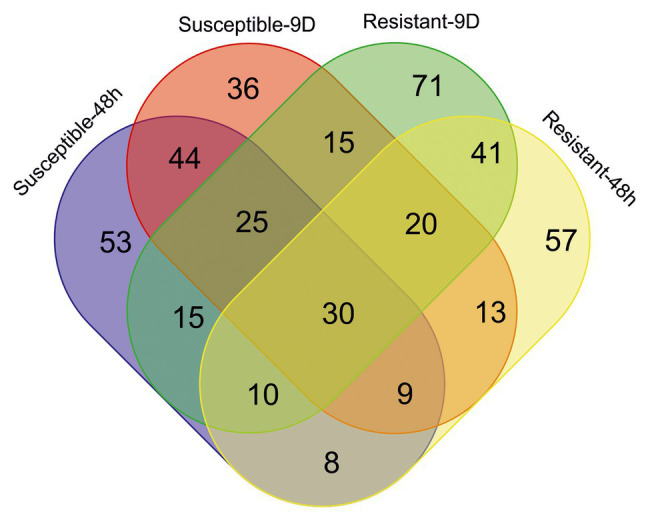
Venn diagram showing the number of important features for each inbred response to each infestation treatment. Ions were considered important for a particular response when their variable importance in projection (VIP) scores in the partial least squares discriminant analysis (PLS-DA) were above 2. Resistant, inbred line PB130; Susceptible, inbred line EP42; 48h, plants pre-infested with Mediterranean corn borer (MCB) larvae for 48h; 9D, plants pre-infested with MCB larvae for 9days.

Attending to the metabolomic approach, 4,362 different features were detected among the different samples (Supplementary Table S1). In the short- and long-term responses, 194 and 192 ions were, respectively, selected as important features (based on VIP scores of the PLS-DA analysis) of the susceptible inbred line, 108 of them being important in both responses (Figure 4). On the other hand, 101 ions out of the 188 and 227 detected as relevant in the respective short- and long-term responses of the resistant inbred were common in both responses. Although 194 and 188 ions had important contributions to the 48-h responses to *S. nonagrioides* in the susceptible and resistant inbreds, respectively, only 57 of them contributed to both responses. Ninety ions were selected as important features based on VIP scores of the PLS-DA analysis for the responses to 9days of stem borer feeding in both inbreds. Finally, 30 ions were involved in the four responses.

A tentative annotation of ions with relevant effects on the long-term response, either detected across genotypes or genotype-specific, was performed (Supplementary Table S2). A general overview of our metabolomic results yields a downregulation of the primary metabolism in both maize genotypes by 9days of MCB feeding. This includes sugar, amino acid, fatty acid, and vitamin metabolism, as well as the tricarboxylic acid (TCA) or Krebs cycle, which implies a disruption of energy production (Liu et al., 2010; Wang et al., 2016; Kang et al., 2019; Sabino et al., 2019). Especially remarkable is that the organic acids malate and malonate were only detected in stems of PB130 preconditioned by 9days of feeding. This could be an indicator of a high redox level of the stem cells in this inbred line. According to that hypothesis, we observed an accumulation of glutathione in the resistant inbred at control conditions, and its levels were doubled in both inbreds after 9days of damage by MCB larvae. In parallel, oxoproline, which is a reservoir of glutamate and participates in glutathione homeostasis (Ohkama-Ohtsu et al., 2008), decreased in the susceptible but augmented in the resistant inbred. Likewise, 3-hydroxy-3-methylglutarate accumulated at high levels in the stem of the resistant inbred after continuous feeding by MCB larvae.

Both genotypes differ on the accumulation of intermediates or end products of the shikimate pathway. Decreases of tryptophan, phenylalanine, and beta-tyrosine levels and those of several phenolic and indole-related compounds derived from them were registered in the susceptible inbred after 9days of feeding. On the contrary, indole-derived DIMBOA glucoside (antibiotic against *S. nonagrioides* larvae) and indole-acrylate (plant hormone) or the phenylpropanoids methyl-4-methoxy-3-nitrobenzoate (probable insecticide), 4-hydroxy-6-methylcoumarin (biocide), sinapaldehyde (intermediate in lignin formation), or o-hydroxyhippurate (insect antifeedant, also known as salicylurate; Ortego et al., 1998; Stuart et al., 2000; Cutler et al., 2002; Feng et al., 2018) were upregulated in the resistant inbred by MCB feeding. We also observed a decrease of several nitrogen-containing compounds in the susceptible inbred, while those compounds increased in the resistant inbred after 9days of feeding; however, levels were still higher in the susceptible inbred.

Other major groups of compounds related to plant resistance to biotic stresses are oxylipins. These compounds are produced by enzymatic or chemical oxygenation of free or membrane-esterified polyunsaturated fatty acids. The major oxylipin identified in plants is jasmonic acid (JA). This phytohormone was upregulated and downregulated by 48h of feeding in resistant and susceptible inbreds, respectively. In parallel, 13-hydroxylinolenic acid (13-HOTrE), which resulted from reducing HPOTrE[R] and is not a JA intermediate, was downregulated by 48h of feeding in the resistant inbred, contributing to the precursor pool and leading to cyclization and eventual synthesis of JA (Farmer et al., 1994). Epoxy and hydroxy derivatives of linoleic acid resulting from the peroxygenase pathway have been described as fungitoxic oxylipins. The metabolite 13-hydroperoxyoctadecadienoic acid, was reduced by 48h of feeding in the resistant inbred and by 9days of feeding in both; meanwhile, vernoleate diminished after 9days of feeding in both.

## Discussion

Previous studies have reported that based on field resistance (always evaluated under artificial infestation), the inbred line PB130 is more resistant to the attack of MCB than the inbred line EP42 (Butron et al., 1999; Ordas et al., 2010). However, in the current study, constitutive antibiosis was higher in the susceptible (EP42) than in the resistant inbred (PB130). These results suggest that induced metabolic responses could have an important role in resistance to stem borers in the long-term period. In agreement with this idea, we observed a disruption of the constitutive defenses in the susceptible inbred because stems from susceptible plants preconditioned for a long period increased larval weights compared to the susceptible control plants, whereas the resistant inbred reduced larval weight compared to the control. Dafoe et al. (2011, 2013) showed that 24–48h feeding by *Ostrinia nubilalis*, the European corn borer, could increase stem susceptibility of a single genotype, but other authors recently reported increased antibiosis of maize leaves infested with *Ostrinia furnacalis*, the Asian corn borer (Guo et al., 2017, 2019). All these results together suggest that inducible rather than constitutive mechanisms have an important role in resistance to stem borers in the long-term period, and alteration of plant performance under subsequent conspecific attack due to previous insect damage is genotype-dependent. Similar results have been previously reported in other plant-insect interactions (Su et al., 2018). For instance, the observed differential defense responses of two different switchgrass cultivars to fall armyworm herbivory indicate that the resistant cultivar mounted a more robust response with potential activation of pathways that could lead to the production of antifeedants, as compared to the susceptible one (Palmer et al., 2019).

These inducible defenses are likely progressive from the initiation of damage because reduction of larval weight in the resistant genotype is stronger in the long-term than in the short-term feeding. We hypothesize that after a prolonged period of insect damage, progressive accumulation of induced defensive metabolites would allow the resistant inbred to perform better than the susceptible inbred against insect feeding, as it has been reported in previous studies (Butron et al., 1999; Cao et al., 2019). Accumulated defenses in the resistant genotype after 9days of insect feeding increased the larval mortality and decreased the larval weight. Nevertheless, these defenses would still not be enough to outperform the antibiosis observed on the susceptible inbred. Even so, better field performance of the resistant inbred against MCB feeding would greatly depend on the accumulation of induced defenses by MCB feeding. This accumulation would be higher as exposure to insect damage is prolonged. Conversely, higher constitutive resistance does not guarantee good performance under MCB infestation because continuous insect damage could disrupt constitutive defenses. These results suggest that the level of field resistance of the studied inbred lines rather depends on induced changes by MCB feeding than on constitutive defenses, and those changes are determined by the duration of insect feeding. Continuous damage by insect feeding seems to contribute to increased susceptibility or resistance, depending on the genotype, and those genotype-dependent changes could be considered the result of reconfiguration of metabolism in attacked plants (Schuman and Baldwin, 2016).

Based on the former hypothesis, maize stems under MCB feeding undergo extensive metabolic reorganization. Those metabolites involved in the long-term response could be important determinants of genotype-induced resistance. As metabolomic analyses were made using undamaged stem sections, herbivore challenged plants of the resistant inbred would be capable of mounting systemically active defense responses; meanwhile, susceptible plants could not (Howe and Jander, 2008). Other authors have demonstrated that maize herbivory by *Mythimna separata* conferred resistance to the subsequently infested caterpillars through systemic changes of benzoxazinoids and probably other defensive metabolites (Malook et al., 2019). Our findings agreed with expectations because plant response to insect attack has been proven to be genotype-dependent, resulting in increased levels of phenylpropanoids, hydroxamic acids, or/and nitrogen-containing secondary metabolites in resistant genotypes. Increased susceptibility after insect attack in other genotypes was previously associated with reduced levels of phenylpropanoids (Liu et al., 2010; Biyiklioglu et al., 2018; Su et al., 2018; Kang et al., 2019). Therefore, the susceptible inbred in field conditions seemed to constitutively possess a resistance metabolic array that is broken down by continuous insect feeding; meanwhile, the field resistant inbred appeared to acquire induced resistance by channeling metabolism toward biosynthesis of defensive metabolites like the benzoxazinoid DIMBOA glucoside and methyl-4-methoxy-3-nitrobenzoate (Schuman and Baldwin, 2016).

Oxylipins has been highlighted as key regulators mediating maize resistance response to herbivory by insects (Christensen et al., 2015; Borrego and Kolomiets, 2016). JA is a 13-LOX α-linolenic acid-derived plant oxylipin. Based on our results, we speculate that maintenance of the upregulation of JA precursors after 48h of feeding in the resistant inbred could play an important role in inducing systemic resistance; meanwhile, resistance was not induced in the susceptible inbred because jasmonate precursors were downregulated. Production of JA is required for a systemic response to herbivory (Bosch et al., 2014). Systemic response to parasites involves transduction processes in which transient shifts of intracellular and apoplastic pH are essential: rapid alkalinization of the apoplast is combined with intracellular acidification, loss of K+, and influx of Ca2+ and followed by an oxidative burst and upregulation of several pathways involved in defense (Viehweger et al., 2002). In that context, authors proposed lysophosphatidylcholines as good candidates for transducing the elicitor-triggered signal; lysophosphatidylcholines would be intracytoplasmic messengers that would connect the activation of a stress-responding enzyme of the plasma membrane (phospholipase A2) with the production of vacuolar proton fluxes. According to that idea, levels of lysophosphatidylcholines augmented in the stems of the resistant inbred after feeding by MCB larvae, while the levels diminished in the susceptible inbred, explaining the increased susceptibility upon feeding in the susceptible inbred. Longer maintenance of high JA precursor levels and upregulation of lysophosphatidylcholines could act as signal molecules of induced systemic resistance (ISR) to stem tunneling by stem borers. ISR would imply an oxidative burst [counterbalanced by increased reactive oxygen species (ROS) scavenging metabolites] and upregulation of metabolites involved in defense.

Accumulation of malate and especially malonate (only detected in stems of PB130 preconditioned by 9days of feeding) in the resistant inbred could be a consequence of a high redox level in the stem cells of this genotype. Under those conditions, the TCA cycle in mitochondria is transformed to a “non-cyclic” partial TCA cycle supplying citrate for the synthesis of 2-oxoglutarate, glutamate, and malonate (citrate valve), while malate is stored and participates in the redox balance (*via* malate valve; Igamberdiev and Eprintsev, 2016). In that scenario, the 3-hydroxy-3-methylglutarate that is an “off-product” intermediate in the leucine degradation process could be proposed as an important agent in causing and maintaining oxidative burst, since this metabolite causes acute disruption of redox homeostasis in animal tissues (da Rosa et al., 2013). Agreeing with the hypothesis of high oxidative stress in the resistant inbred after 9days of feeding by MCB larvae, this inbred line presents a high ROS scavenging level that increased after long-term feeding, as indicated by the accumulation of glutathione and oxoproline, in the resistant inbred.

## Conclusion

We hypothesize that the level of field resistance depends on induced changes by MCB feeding rather than on constitutive defenses, and those changes are determined by the duration of insect feeding. Therefore, differential defense responses to continuous MCB feeding would result in resistance differences and ions that were differentially induced in both inbreds by long-term feeding could play an important role in resistance. A limited number of differentially induced features could be assigned to known metabolites but point to the ability of the resistant inbred to establish a systemic response involving oxidative burst and upregulation of defense compounds as determinants for limiting damage by MCB larvae. Therefore, these results encourage a high throughput look for specific metabolites implicated in systemic induced resistance to maize stem borers instead of the current focus on constitutive defense metabolites. Those molecules would be a valuable tool for pest control in those scenarios where transgenic crops are not allowed, such as organic agriculture.

## Data Availability Statement

The raw data supporting the conclusions of this article will be made available by the authors, without undue reservation.

## Author Contributions

AB and RM conceived and designed the research. PV, VR, AC, RS, and AB conducted the experiments. AB, PV, and VR analyzed the data. AB wrote the manuscript. All authors contributed to the article and approved the submitted version.

### Conflict of Interest

The authors declare that the research was conducted in the absence of any commercial or financial relationships that could be construed as a potential conflict of interest.
